# Experimental data of bio self-healing concrete incubated in saturated natural soil

**DOI:** 10.1016/j.dib.2019.104394

**Published:** 2019-08-22

**Authors:** Adam Souid, Mohamed Esaker, David Elliott, Omar Hamza

**Affiliations:** a Marie Sklodowska-Curie Research Fellow, College of Engineering and Technology, University of Derby, UK; b College of Engineering and Technology, University of Derby, UK; c College of Natural and Life Science, University of Derby, UK; d College of Engineering and Technology, University of Derby, Derby, DE22 3AW, UK

**Keywords:** Self-healing concrete, Bacteria, Soil incubation, Scanning Electron Microscope (SEM), Energy Dispersive X-Ray Spectrometry (EDX)

## Abstract

The provision of suitable incubation environments is vital for successful implementation of bio self-healing concrete (bio-concrete). We investigated the effect of soil incubation to examine if the self-healing process can be activated in comparison with the conventional incubation environment (water). The data was collected from laboratory-scale experiments conducted on mortar specimens. The mortar was impregnated with *Bacillus subtilis* and this bacteria was encapsulated in calcium alginate for protection from the production process. The mortar specimens were mechanically cracked and then incubated within fine-grained fully saturated natural soil for about 4 weeks. The cracks were inspected before and after incubation by light microscopy to evaluate the healing ratio. The mineral precipitations on crack surfaces were examined by Scanning Electron Microscope (SEM) and Energy Dispersive X-Ray Spectrometry (EDX). The data reflects the efficiency of bio-concrete for certain structures such as tunnels and deep foundation, where concrete elements are exposed to ground conditions.

**Table:** Specifications Table

Subject	Civil and Structural Engineering
Specific subject area	Construction materials and concrete
Type of data	Table, Figure, Image
How data were acquired	Mainly by a series of laboratory-scale experiments using: Prismatic mortar specimens (produced in the laboratory) *Bacillus subtilis* H50620/9 (supplied by Philip Harris, UK) Natural soil (locally resources) Mortar Mixer (CONTROLS) Microscope (LABOPHOT-2, Nikon) Compression machine (CONTROLS) Scanning Electron Microscope (SEM) Energy Dispersive X-Ray Spectrometry (EDX) Miniature-Tensiometer (UMS T5)
Data format	Raw and Analyzed
Parameters for data collection	The provision of bacterial self-healing agent within specimens (with and without). The incubation environment surrounding the specimens (soil and water).
Description of data collection	Mortar specimens (with and without bacterial self-healing agent) were cast, cured for 28 days, initially cracked and then incubated either in saturated soil or water for 4 weeks. Cracks were inspected before and after the incubation to evaluate the self-healing.
Data source location	College of Engineering and Technology, University of Derby, UK
Data accessibility	Data is provided within the article

**Table:** 

**Value of the data** • This experimental data examines if the bio self-healing concrete can work in soil environment. • The data allows for the comparison between healing ratio of specimens incubated in saturated natural soil and specimens incubated in conventional environment i.e. water. • The data helps to determine if the soil's bacteria are able to heal the concrete cracks in a natural way i.e. without adding any bacterial self-healing agent to the concrete mixture. • The data can benefit engineers and scientists who are involved with the design and development of resilient construction materials for underground structures. • The data provides a good basis for further research to explore the effect of different environmental exposures posed by soil on the bio self-healing process.

## 1 Data

**Fig. 1:**
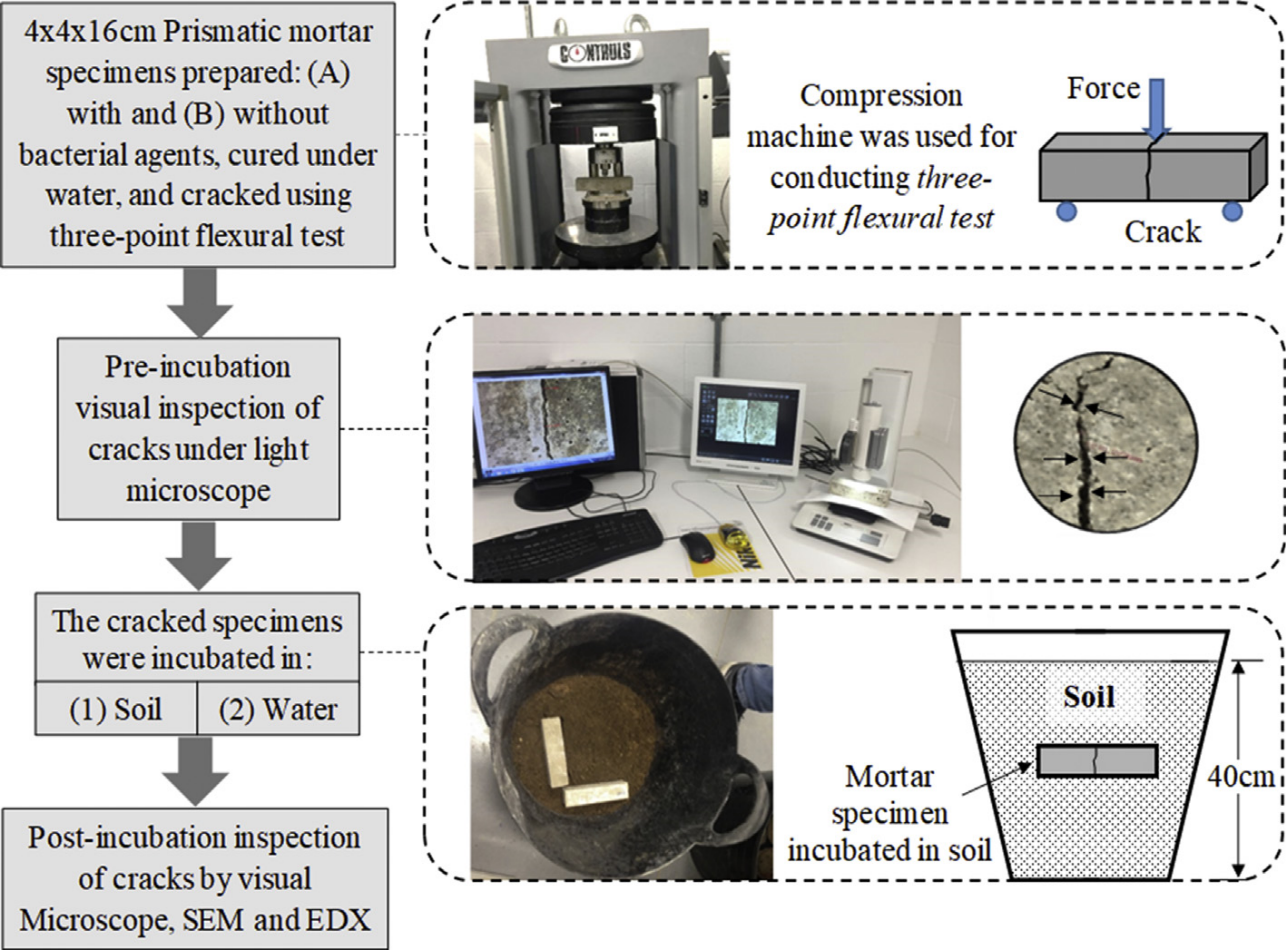
A general overview of the method adopted for the experimental work and how data was collected.

**Fig. 2:**
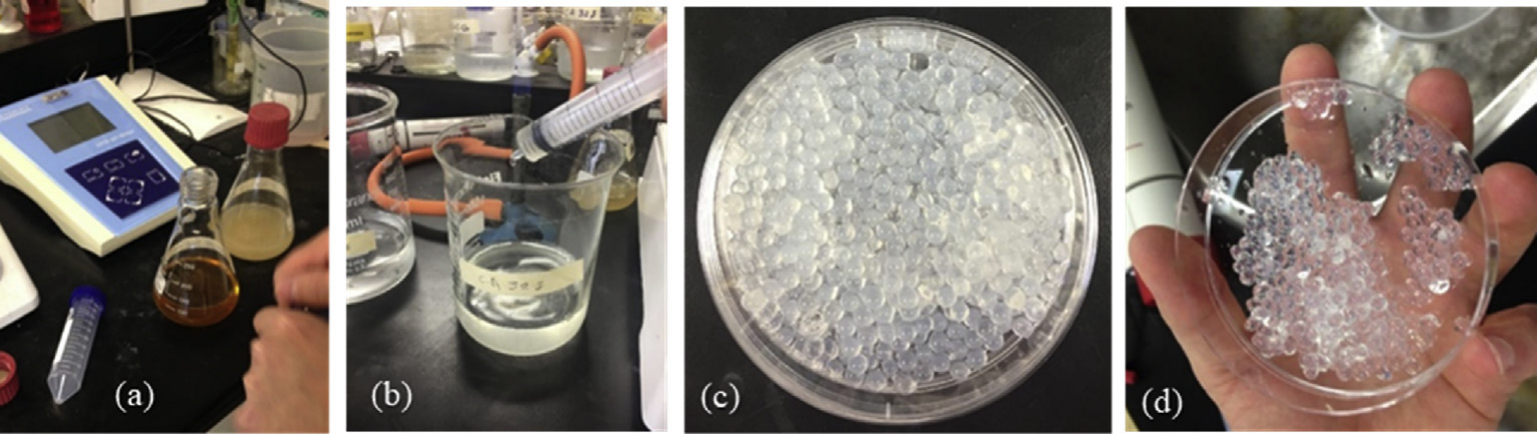
Experimental procedures: (a) obtaining spores from bacterial culturing solution after 20 hours in an incubator at 37 °C, (a) Encapsulation process of spores within Calcium Alginate, (b) and (d) the Calcium Alginate Beads (CAB).

**Fig. 3:**
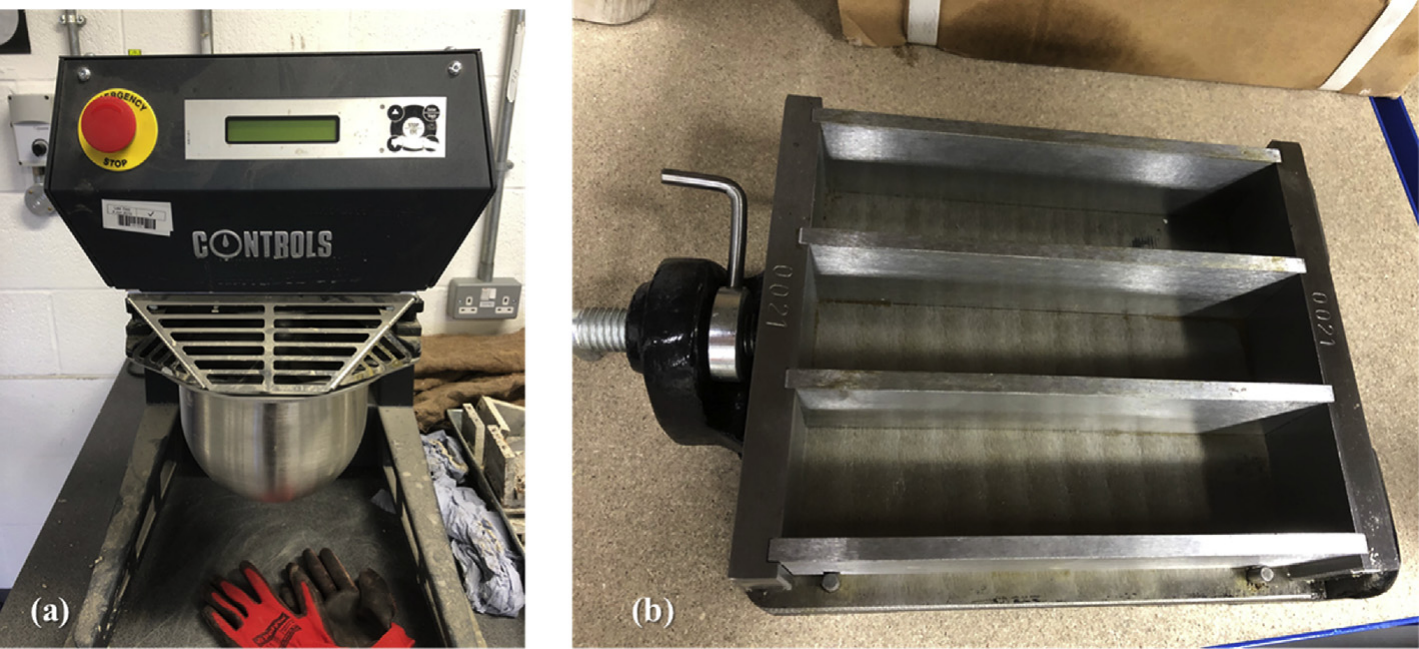
(a) Mixer used to prepare the mortar, (b) Steel mould with a capacity of three specimens.

**Fig. 4:**
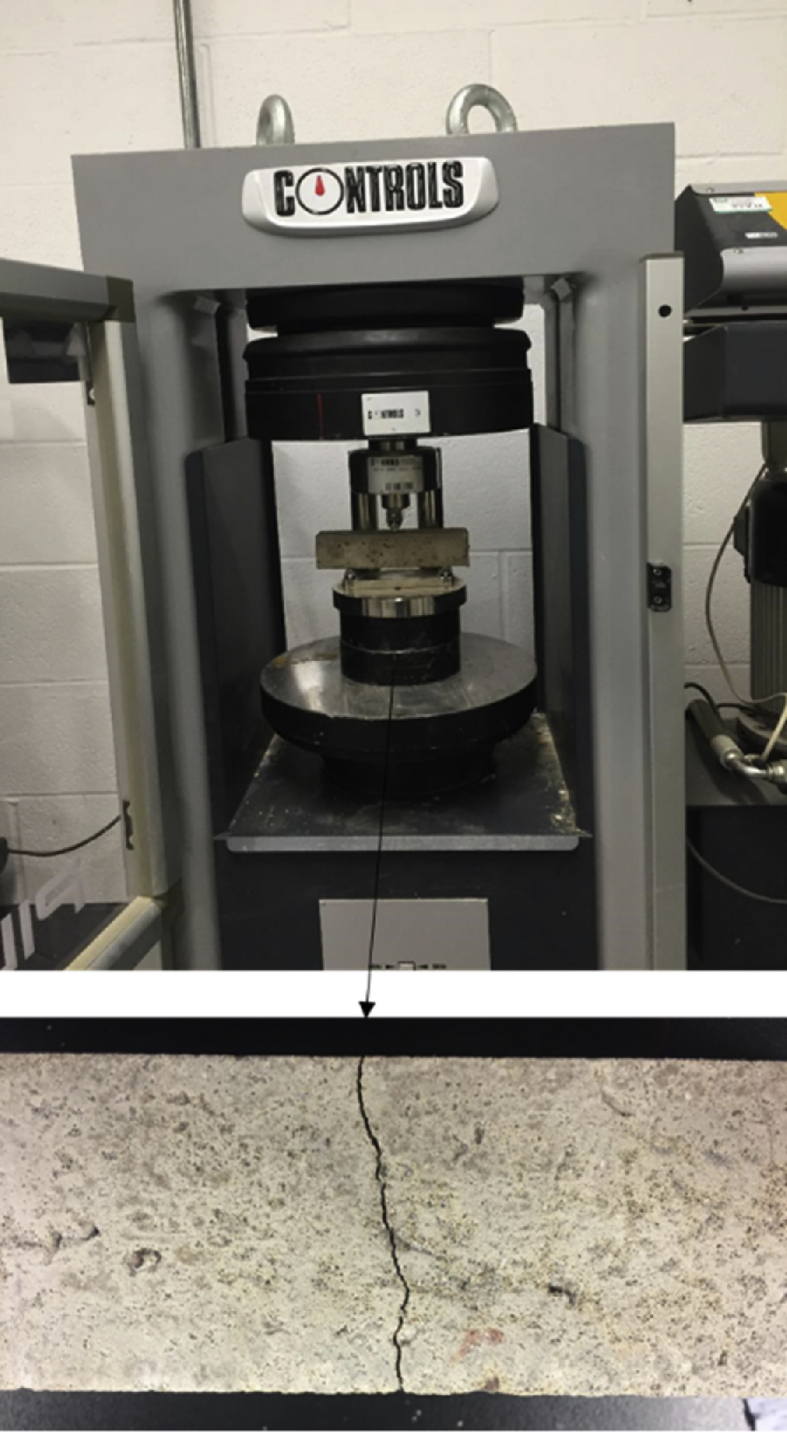
Three-point bending test used to generate a crack in the middle of specimens.

**Fig. 5:**
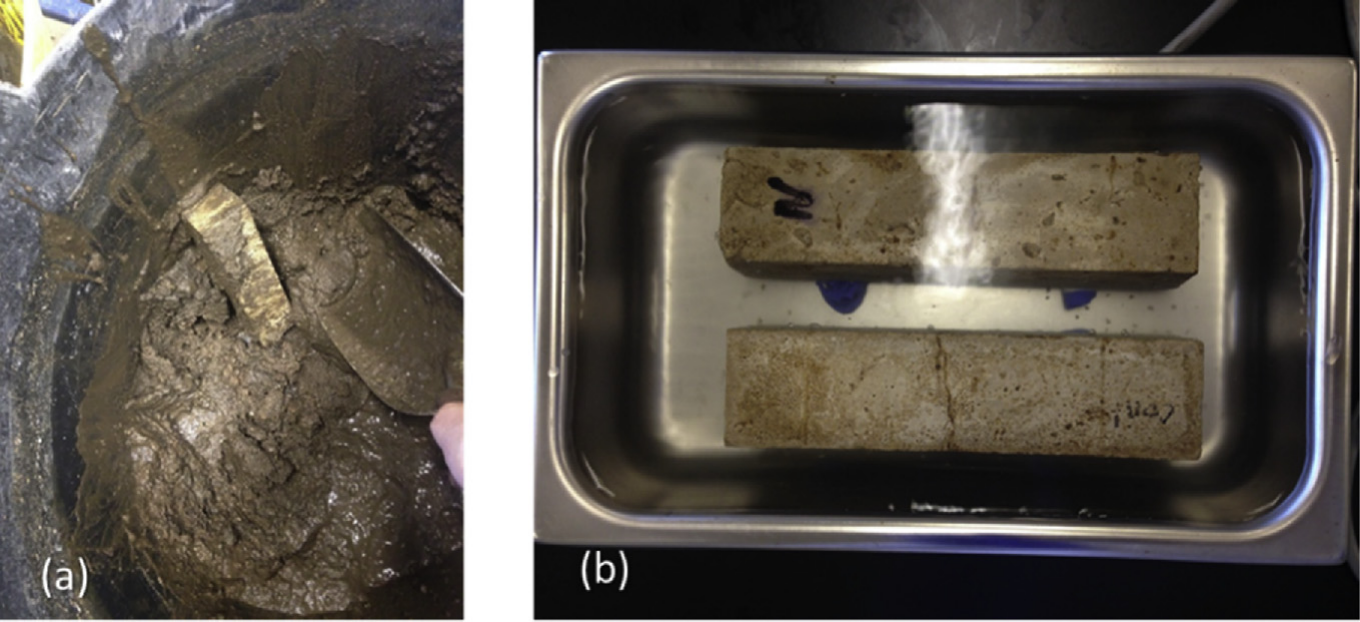
(a) Photo taken while recovering a specimen from the soil, (b) Ultrasound cleaning in water to remove any remaining soil particles covering the specimens.

The data was collected from laboratory experimental programme conducted on mortar specimens. This data is presented in a total of 4 tables and 8 figures. Fig. 1 shows a general overview of the steps adopted for the experimental work and how data was collected while testing different types of mortar specimens. Table 1, Table 2 present the data of these mortar specimens including the number of specimens, testing conditions and the materials used to produce them. Fig. 2, Fig. 3, Fig. 4, Fig. 5 are photos taken during both material preparation in the microbiology laboratory and specimens’ testing in the concrete laboratory.

**Table 1:** Types of mortar specimens used for the experiments.

Specimens ID	Inculpation medium	Bacterial agent	Purpose	Quantity
S1-A	Saturated soil	Added	Main test	3
S1–B	Saturated soil	Not added	Control test	3
S2-A	Tap water	Added	Main test	3
S2–B	Tap water	Not added	Control test	3

**Table 2:** Mixture proportions (mass ratio) of the mortar specimens.

Specimen type	Self-healing agent∗:Cement	Sand:Cement	Water:Cement
S1-A, S2-A	0.25	3.25	0.5
S1–B, S2–B	0	3.25	0.5

∗ Bacteria encapsulated in Calcium alginate beads.

**Fig. 6:**
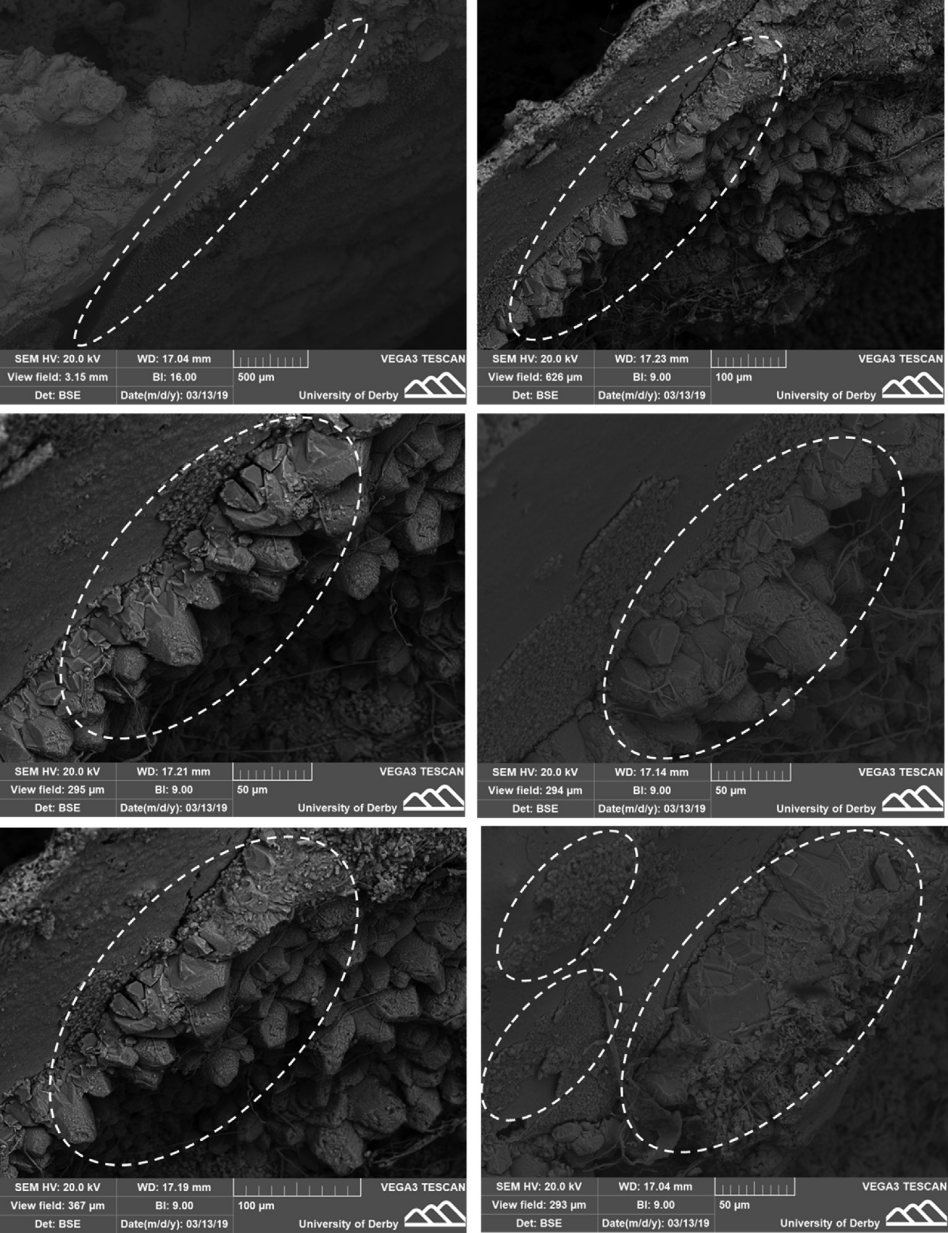
SEM data taken from soil-inculpated specimens (S1-A) showing zones of precipitates at different scales and locations.

**Fig. 7:**
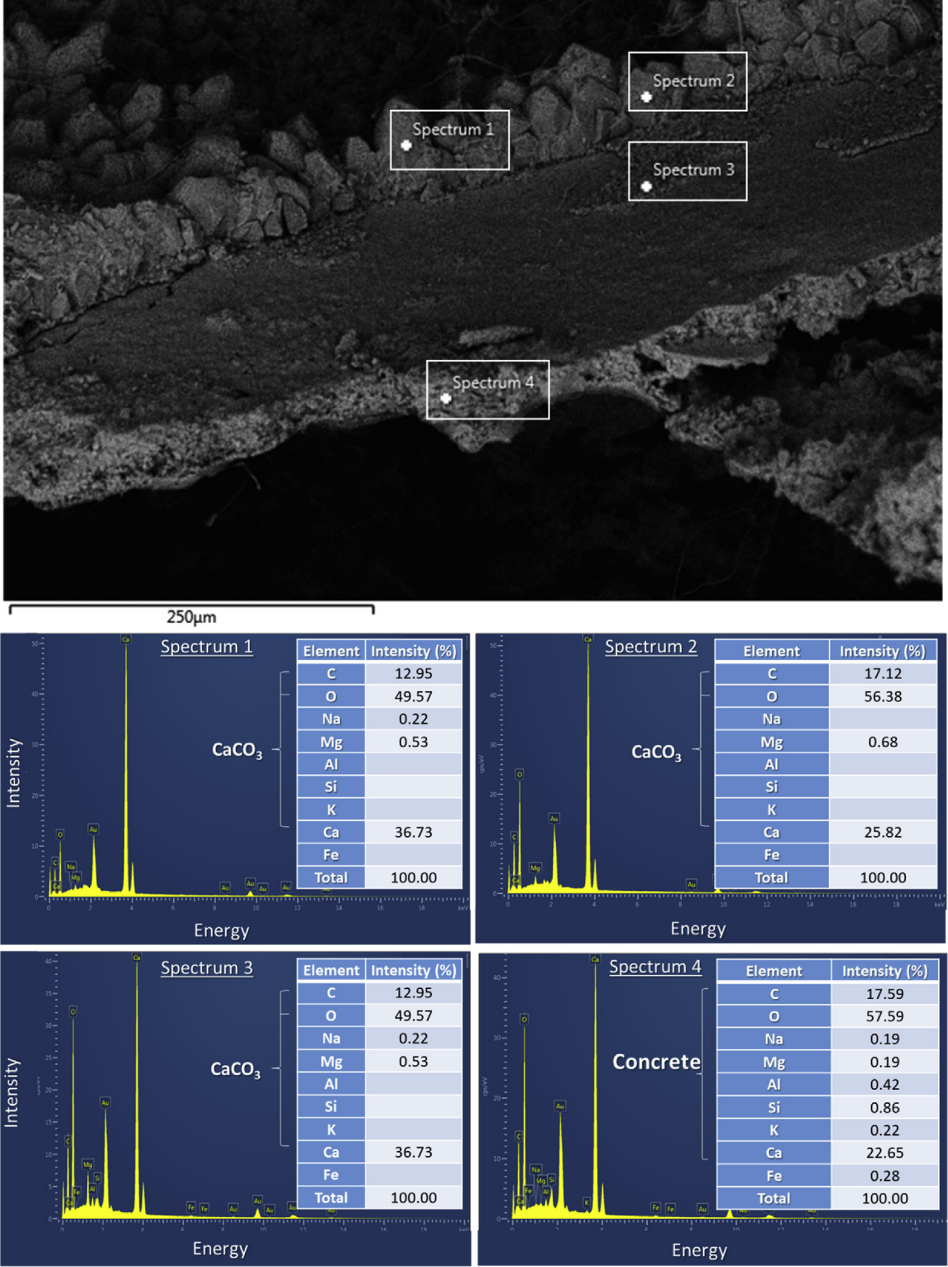
EDX data obtained from soil-inculpated specimen (type S1-A) at different spectrums located: within the materials precipitated at the crack edge (spectrums 1, 2, 3), and within the concrete (spectrum 4).

**Fig. 8:**
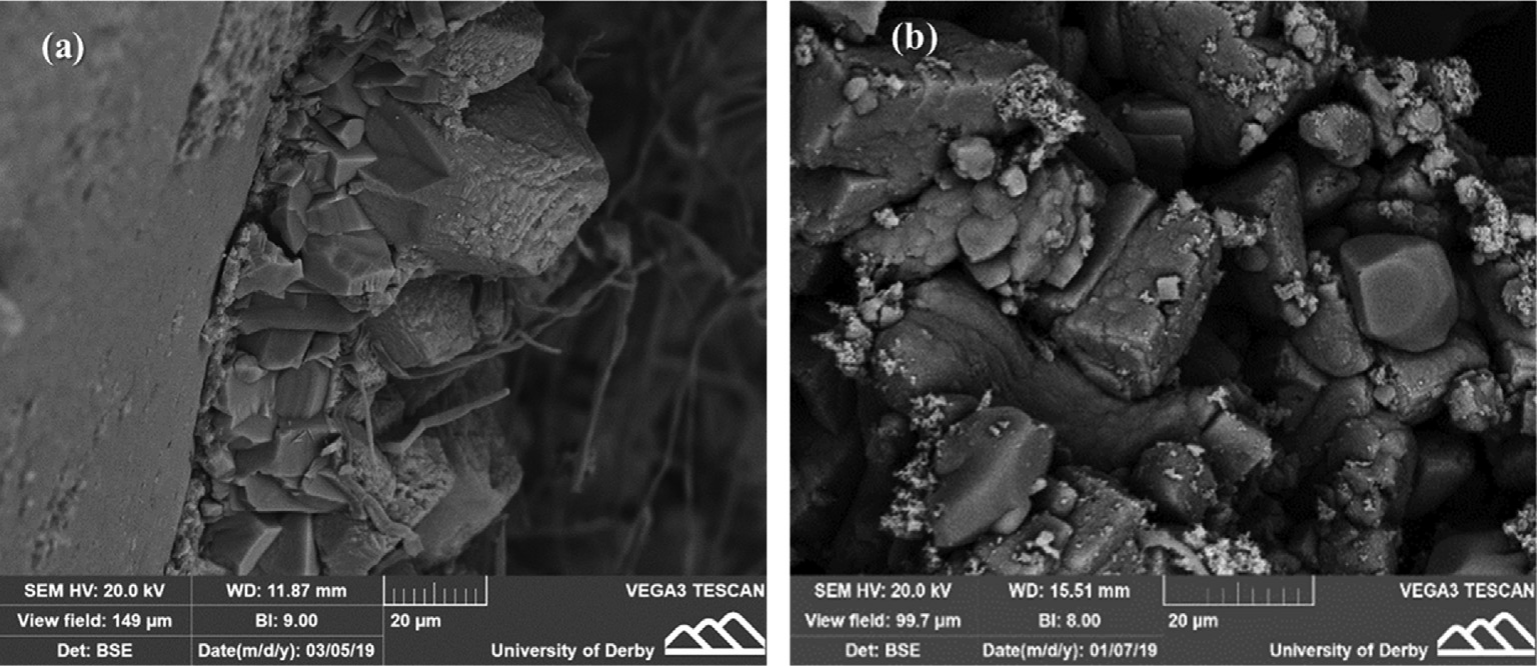
SEM data of the precipitated product in two healed cracks taken from (a) Soil—incubated specimen S1-A and (b) Water-incubated specimen S2-A.

Fig. 6, Fig. 8 show the data collected from the Scanning Electron Microscope (SEM) conducted on different zones of the specimens' cracks (including the mineral precipitations), while Fig. 7 presents the chemical composition data of these scanned zones using Energy Dispersive X-Ray Spectrometry (EDX). Table 3, Table 4 present the experimental data measured and observed during the experiments including the negative pore water pressure in the soil (Table 3) and the healing developed in the mortars’ cracks (Table 4).

**Table 3:** Tensiometer data of the soil suction.

Time (day)	Suction (kPa)	Time (day)	Suction (kPa)
0	0.2	17	0.5
3	0.1	19	0.6
6	0.03	21	1.9
9	0.6	24	1.7
12	0.1	27	4.3
15	0.9	28	4.1

**Table 4:** Crack healing observed in different specimens.

Specimens ID	Inculpation medium	Bacterial agent	Observation of crack healing
S1-A	Saturated soil	Added	Next highest
S1–B	Saturated soil	Not added	The least
S2-A	Tap water	Added	The highest
S2–B	Tap water	Not added	A slight

## 2 Experimental design, materials, and methods

The bio-concrete has previously been examined in water and humid air [1], [2], [3]. Our research work explored the application of this innovative technology in soil environment. This experimental work was conducted to examine if the bio self-healing process can be activated within pre-cracked mortar specimens under saturated soil and whether the healing efficiency is comparable with conventional incubation environment i.e. within water.

The work was achieved through the development of a novel experimental approach combining several technologies applied in materials/concrete, geotechnical engineering, and microbiology. The experiments were conducted on twelve mortar specimens as explained in Table 1. Some of the experiments served as control tests.

The conceptual design and method adopted for the experimental work are graphically shown in Fig. 1. These are explained in more details in the next following sections.

### 2.1 Preparation of bacterial agent/Encapsulation

Many types of bacteria have been used in self-healing concrete studies by different researchers. Our work considered *Bacillus subtilis* H50620/9 because of its ability to form high-resistant long-lived spores [4], [5]. The process of preparing the bacterial-based self-healing agent started by cultivating bacterial strains. These were conducted in Basal medium 121 including its derivatives 121A and 121B in a similar approach adopted by Sonenshein et al. [6]. The culture was incubated in a shaker at 120 rpm at a temperature of 36 °C for 72 hours until the formation of spores was observed. Spore formation was confirmed under a light microscope using the Gram Staining Method (GSM) [7]. To minimize the presence of vegetative cells, spores were harvested with the use of a centrifuge machine, where the culture was spun at very high speed (3390 RCF) for 10 min and then washed twice using distilled water. The centrifugal force causes heavier particles to move away from the axis of rotation, resulting in the deposition of spores at the bottom of the test tube forming what is known by a pellet.

Calcium Alginate (CA) was used as a carrier (capsules) for the bacterial spores and nutrients [8]. The capsules were prepared by making a solution consisting of 7.5 g of sodium alginate, 0.5 g of yeast extract (nutrient), 7.8 g of hydrochloride (as alkali buffer - 0.1 mol. l^−1^) and bacterial spores (6.1 × 10^6^ CFU. ml^−1^). In this research work, the serial dilutions method has been applied to estimate the number of bacterial colonies according to Nakahata and Ogawa [9]. The solution was mixed homogeneously using a magnetic stirrer to form a 1.5% bacterial sodium alginate solution. Fig. 2 shows some photos taken during this preparation stage.

To form spherical capsules of calcium alginate, the solution was dropped by syringe (Fig. 2-b) into a coagulate solution. This solution consisted of 8 g of calcium chloride and 4 g of calcium lactate mixed in 400 ml of sterilized water. After about 20 minutes, the formed beads were removed from the calcium chloride solution, washed twice using sterilized water, and dried at 37 °C for 24 h. The particle size of the produced capsules was approximately 150 μm (Fig. 2-c and 2-d).

### 2.2 Mortar specimen – materials and preparation

Prism specimens (with dimensions of 4x4x16 cm) were prepared by mixing mortar components and casting the mixture in steel moulds. Each specimen was reinforced by a single axial steel bar to prevent full breakage during the creation of crack in the next stage.

The first component used in the specimens’ mixture was Portland cement (CEM II/B–V 32.5R), which is a commercially available product for making conventional concrete, mortars, renders, screeds and grouts. In addition to the cement, the mixture contained fine aggregate sand, and tap water in accordance with the mixture proportions indicated in Table 2. The fine aggregate sand (with a maximum particle size of 2 mm, and a specific gravity of 2.63) was sourced from Travis Perkins in the UK. Any moisture in the sand was removed by heating in an oven at 105 °C overnight to avoid the effect of the sand moisture on the water-cement ratio.

After weighing out all the mix ingredients in the correct proportions by using a digital scale, the cement mortar specimens were prepared by mixing the cement with water in a digital mortar mixer (Fig. 3a) conforming to BS EN 196-1 [10] for about 30 seconds at the low speed (140 ± 5 r/m). The sand was added gradually to the cement paste and the mixing continued for 30 seconds with high speed (285 ± 10). The mixer was stopped for 30 seconds to scrape any adhering paste from the mixer's walls, and then the mix was left to rest for 60 seconds. The mixer was switched on again at high speed and continued mixing for an additional 60 seconds.

The selected bacterial spores and nutrient (encapsulated in calcium alginate beads) were added in the final stage before casting the concrete mixture in the moulds. The mortar was then hand compacted into the steel mould, which has a capacity of three specimens as shown in Fig. 3b. To remove any trapped air, the mould was placed on a vibrating table for about 5 minutes. At the end of this stage, the mortar (in the mould) was covered with a damp cloth to avoid surface dryness and left overnight to harden. After 24 hrs the mortar specimens were hard enough to be removed from their mould without any damage. The speciemns were immersed in a tap water for a curing period of 28 days to allow the material to gain its strength [10]. The procedures explained above were generally adopted for all mortar specimens to maintain consisitancy.

### 2.3 Specimen cracks - mechanical generation and visual inspection

After the curing period, the specimens were removed from the water and placed in a compression machine to create a crack. A single crack was created around the middle of each specimen by conducting a three-point flexural test as shown in Fig. 4.

In this test, the prism was installed at two parallel beams at the bottom of the sample and the distance between these beams was around two-thirds of the sample's total length. The top surface of the sample was compressed by one central beam as shown in Fig. 4. The load was applied gradually with a velocity of 0.5 m/s until a crack was formed. At that point, the velocity was decreased to allow the crack to be formed around the specimen without failing it, and then the specimen was unloaded giving cause to a decrease in crack width.

Specimens were carefully removed from the compression machine to measure the initial widths of the cracks under the light microscope. The average value of the initial crack width for all specimens was approximately 0.3mm.

### 2.4 Incubation in soil and water

Incubating the mortar specimens in a suitable environment for a period of time is necessary to activate the self-healing process and sealing the crack. For this study, the specimens were split into four groups (S1 and S2) as presented in Table 1. The first group S1 (half of the total number of specimens) were incubated in saturated soil for 4 weeks. To serve as a control test, the other group (S2) were incubated in tap water for a typical duration i.e. 4 weeks. In addition, some of the specimens (S1–B and S2–B) were not impregnated with bacterial agent in order to examine any natural healing that could be developed by the aid of the naturally existing soil bacteria or by the ongoing hydration within the mortars.

The soil was locally resourced from a natural field within the University of Derby. The soil was first examined visually to determine its class. It was classified as natural alluvial soft to firm dark brown silty slightly sandy clay. This alluvial clay would contain a wide range of bacteria naturally existed within the ground. The soil had neutral pH values of 6.5–7.6, measured by a hand-held soil pH tester. A similar range of pH was also observed for the tap water used for incubating the other groups of specimens.

Further analysis of the soil using Particle Size Distribution testing indicated a very high content of fine-grained material (more than 35% of clay) and less than 7.4% sand and gravel. As the fine-grained material is highly impermeable, an additional amount (about 15%) of medium-grained sand was added to the soil to slightly improve its permeability.

The soil incubation was conducted within plastic containers as shown in Fig. 1. Each container hosted three specimens of the same type. The initial condition of the soil was made fully saturated where negative pore water pressure (suction) should be almost 0. This condition was confirmed by measuring the soil suction using Miniature-Tensiometer (UMS T5). The measured data of the soil suction during the test are shown in Table 3.

### 2.5 Post-incubation crack inspection and scanning

At the end of the incubation period, all specimens were removed from their incubation environments (soil and water) to measure the crack widths again and quantify the efficiency of bio-self healing. In preparation for the cracks' inspection (particularly for the specimens incubated in soil), it was essential to conduct ultrasound cleaning (within water) to remove any remaining soil particles (Fig. 5). These specimens were then dried and broken along their cracks to scan the cracks’ lips.

The cracks were first visually inspected under the light microscope. This step was further supported by Scanning Electron Microscope (SEM) to check the microstructure of the cracks any material precipitated (i.e. calcium carbonate). The SEM data is shown in Fig. 6 taken from specimens S1-A.

The chemical compositions of the precipitated materials, as well as the concrete (mortar), were examined by Energy Dispersive X-Ray Spectrometry (EDX) as shown in Fig. 7. The EDX analysis confirmed the precipitated materials had a signature of calcium carbonate (CaCO_3_). This crack sealing material was largely observed in the specimens impregnated with bacterial agents. Summary of the crack healing observation is provided in Table 4.

Different crystal sizes and shapes of hexagonal structure, and other phases with spherical shapes of the calcium carbonate were observed in both types of specimens S1-A and S2-A as shown in Fig. 8.
